# Genetic polymorphisms of DNA double-strand break repair pathway genes and glioma susceptibility

**DOI:** 10.1186/1471-2407-13-234

**Published:** 2013-05-10

**Authors:** Peng Zhao, Peng Zou, Lin Zhao, Wei Yan, Chunsheng Kang, Tao Jiang, Yongping You

**Affiliations:** 1 Department of Neurosurgery, the First Affiliated Hospital of Nanjing Medical University, Nanjing, 210029, China; 2 Department of Neurosurgery, Tiantan Hospital, Capital Medical University, Beijing, 100050, China; 3 Department of Neurosurgery, Tianjin Medical University General Hospital, Tianjin, 300052, China

**Keywords:** DNA double-strand breaks (DSBs), Single nucleotide polymorphisms (SNPs), Glioma, Susceptibility

## Abstract

**Background:**

Genetic variations in DNA double-strand break repair genes can influence the ability of a cell to repair damaged DNA and alter an individual’s susceptibility to cancer. We studied whether polymorphisms in DNA double-strand break repair genes are associated with an increased risk of glioma development.

**Methods:**

We genotyped 10 potentially functional single nucleotide polymorphisms (SNPs) in 7 DNA double-strand break repair pathway genes (*XRCC3*, *BRCA2*, *RAG1*, *XRCC5*, *LIG4*, *XRCC4* and *ATM*) in a case–control study including 384 glioma patients and 384 cancer-free controls in a Chinese Han population. Genotypes were determined using the OpenArray platform.

**Results:**

In the single-locus analysis there was a significant association between gliomas and the *LIG4* rs1805388 (Ex2 +54C>T, Thr9Ile) TT genotype (adjusted OR, 3.27; 95% CI, 1.87-5.71), as well as the TC genotype (adjusted OR, 1.62; 95% CI, 1.20-2.18). We also found that the homozygous variant genotype (GG) of *XRCC4* rs1805377 (IVS7-1A>G, splice-site) was associated with a significantly increased risk of gliomas (OR, 1.77; 95% CI, 1.12-2.80). Interestingly, we detected a significant additive and multiplicative interaction effect between the *LIG4* rs1805388 and *XRCC4* rs1805377 polymorphisms with an increasing risk of gliomas. When we stratified our analysis by smoking status, *LIG4* rs1805388 was associated with an increased glioma risk among smokers.

**Conclusions:**

These results indicate for the first time that *LIG4* rs1805388 and *XRCC4* rs1805377, alone or in combination, are associated with a risk of gliomas.

## Background

Gliomas are the most common type of primary intracerebral neoplasm in China as well as in the West, and comprise more than 40% of primary brain tumors in humans [1-3]. Although the etiology of gliomas remains unclear, exposure to ionizing radiation (IR) and genetic alterations are unequivocally associated with an increased risk of gliomas [4].

DNA double-strand breaks (DSBs) can be generated during V(D)J recombination, class-switch recombination at the immunoglobulin heavy chain (IgH) locus or meiosis and result from a variety of factors including ionizing radiation and reactive oxygen species [5]. Inadequacy or defects in DSB repair can lead to large-scale loss of genetic information and can have disastrous consequences such as genomic instability, immunodeficiency, radiosensitivity, cell death and oncogenic transformation [6,7]. DSBs are sensed by the MRN (MRE11, RAD50, and NBS1) complex, which catalyzes activation of ATM [8,9]. Two major pathways have evolved in mammalian cells to repair DSBs: non-homologous end-joining (NHEJ) and homologous recombination (HR). The central components of the NHEJ pathway are Ku70 (XRCC6), Ku80 (XRCC5), DNA-dependent protein kinase catalytic subunit (DNA-PKcs), XRCC4 and DNA ligase IV (LIG4) proteins [10]. RAD51 interacts with other important repair proteins, including BRCA1, BRCA2, XRCC2, and XRCC3 and plays a central role in the HR activation through the use of sister-chromatid sequences as a template for precise repair [11].

Recent evidence suggests that several single nucleotide polymorphisms (SNPs) in the DSB repair pathway genes may be prognostic biomarkers for GBM survival and modulate gamma-radiation-induced mutagen sensitivity in glioma patients [12,13]. Genetic variants in DSB repair pathway genes have been extensively studied in multiple cancers. However, few studies have specifically identified any association between genetic variations in the DSB repair pathway genes and the risk of gliomas. Here we investigate the role of 10 potential SNPs in *XRCC3*, *BRCA2*, *RAG1*, *XRCC5*, *LIG4*, *XRCC4* and *ATM* in the development of gliomas, and further evaluate their gene-gene and gene-environment interactions in the development of glioma.

## Methods

### Study population

The study was approved by the Ethics Review Board of Nanjing Medical University. All studies involving human subjects were conducted under full compliance with government policies and the Helsinki Declaration. From 2005 to 2010, a total of 447 patients with histopathologically confirmed gliomas were recruited from the Department of Neurosurgery of Jiangsu Province Hospital (the First Affiliated Hospital of Nanjing Medical University) and Beijing Tiantan Hospital Neurosurgery Center (BTHNC). The tumors were graded according to the World Health Organization (WHO) classification [3]. A total of 408 (87%) patients agreed to the study. The 400 healthy control subjects without a history of cancer were recruited from health examination clinics at these hospitals concurrent with the recruitment of glioma patients. The control subjects were frequently matched to cases by age and sex. All cases and controls in this study were genetically unrelated ethnic Han Chinese. All participants completed an informed consent in accordance with the requirements of the institutional review board of each participating institution and a structured questionnaire to obtain detailed information on diet, weight, height, smoking habits and drinking status. After the interview a blood sample (5 ml) was collected from each study subject, gathered into heparinized tubes and stored at −70°C until used for DNA extraction and genotyping. Finally, 384 glioma patients and 384 cancer-free controls whose DNA samples were available and adequate were included in our work.

### SNP selection and genotyping

Potential functional SNPs within each gene were identified through an extensive mining of the databases of the International HapMap Project and dbSNP. As a result, 10 SNPs in the coding sequence, promoter, splice sites, and 5^′^ or 3^′^-UTRs with a minor allele frequency (MAF) ≥ 0.05 in the general Han Chinese population were selected.

Genomic DNA was isolated from leukocyte pellets of venous blood by proteinase K digestion and phenol-chloroform extraction. Genotyping was performed using the OpenArray platform (Applied Biosystems, Foster City, California, USA). This platform employs a chip-based TaqMan genotyping technology. Sequences of primers and probes are available on request. Genotyping was conducted according to the manufacturer’s standard protocols, and genotype calls were made by OpenArray SNP Genotyping Analysis Software V.1.0.3. To ensure quality control, genotyping was performed without the knowledge of individual case–control status. We randomly selected 5% of the samples to be genotyped again by different investigators and the reproducibility rate was 100%. To validate the genotyping results, selected PCR-amplified DNA samples (n = 2, for each genotype) were confirmed by DNA sequencing, and these results were found to be 100% concordant.

### Statistical analysis

Demographic variables between cases and controls were compared using Student’s *t* test (age and pack-years) for continuous variables and the χ^2^ test for categorical variables (gender, smoking and drinking status). The Hardy-Weinberg equilibrium (HWE) was evaluated in control subjects using a goodness-of-fit χ^2^ test with 1 degree of freedom. The effect of each SNP on glioma risk was estimated as an odds ratio and 95% confidence intervals (95% CI) by unconditional logistic regression adjusted by age, gender, smoking and drinking status. A score test of linear trend was conducted for each SNP using a three-level ordinal variable. To minimize false positive results generated from the multiple statistical tests used in our analysis, we applied a false discovery rate (FDR) method to the *P* values for trend [14]. To evaluate effect modification by smoking, subgroup analyses were also performed for *LIG4* rs1805388 and *XRCC4* rs1805377 polymorphisms. A more-than-multiplicative gene-gene or gene-environment interaction was evaluated using logistic regression analysis. When the test for multiplicative interaction was not rejected, further test for an additive interaction was done by a bootstrapping test of goodness-of-fit of the null hypothesis, for no departure from the additive model compared to an alternative hypothesis, and for a departure from an additive model by using Stata software (version 8.2; StataCorp LP). All the other statistical analyses were performed with SAS 9.1.3 software (SAS Institute).

## Results

### Sample characteristics

The distribution of demographic characteristics of the 384 cancer cases and 384 cancer-free controls available for this analysis are summarized in Table 1. Cases and controls were well matched on age (*P* = 0.277), gender (*P* = 0.715), smoking status (*P* = 0.465) and drinking status (*P* = 0.222), suggesting that our frequency matching of the demographic characteristics was satisfactory.

**Table 1:** Distribution of selected host characteristics by case–control status in Chinese

**Variables**	**Case (n = 384)**	**Control (n = 384)**	***P***^***b***^
Age, y (mean ± SD)	62.4 ± 10.8	61.5 ± 12.1	0.277
Gender, no. (%)			
Male	222 (57.8)	217 (56.5)	0.715
Female	162 (42.2)	167 (43.5)	
Smoking status, no. (%)			
No	228 (59.4)	218 (56.8)	0.465
Yes	156 (40.6)	166 (43.2)	
Pack-years (mean ± SD)^*a*^	32.7 ± 25.1	30.3 ± 27.7	0.209
Drinking status, no. (%)			
No	288 (75.0)	273 (71.1)	0.222
Yes	96 (25.0)	111 (28.9)	
WHO grade, no. (%)			
WHO I	41 (10.7)		
WHO II	176 (45.8)		
WHO III	86 (22.4)		
WHO IV	81 (21.1)		

^a^Among ever smokers.

^b^*P* values were derived from the χ^2^ test for categorical variables (gender, smoking and drinking status) and t test for continuous variables (age and pack-years).

### Individual SNP association analysis

Primary information of the 10 functional SNPs found in the Chinese population in the dbSNP database is presented in Table 2. All tested SNPs were in agreement with Hardy-Weinberg equilibrium in the control subjects (*p* > 0.05).

**Table 2:** Primary information for 7 genotyped SNPs in DNA repair genes

**Genotyped SNPs**	**Location/or amino acid change**	**MAF for Chinese in database**^**a**^	***P*value for HWE test**
*XRCC3:* rs861539 *C>T*	nsSNP/ T241M	0.067	0.165
*XRCC3*: rs1799794 G>A	5' UTR	0.478	0.271
*XRCC3:* rs1799796 A>T	nsSNP/ A17893G	0.289	0.406
*BRCA2*: rs1799943 G>A	Promoter	0.279	0.722
*BRCA2*: rs15869 A>C	3' UTR	0.244	0.375
*RAG1*: rs2227973 G>A	nsSNP/ R820K	0.465	0.586
*XRCC5*: rs1051685 A>G	3' UTR	0.067	0.808
*LIG4:* rs1805388 *C>T*	nsSNP/T9I	0.261	0.659
*XRCC4*: rs1805377 A>G	Splice Site	0.300	0.454
*ATM*: rs189037 G>A	Promoter	0.389	0.070

MAF, minor allele frequency; HWE, Hardy-Weinberg equilibrium.

^a^Minor allele frequency in the Chinese (CHB, Han Chinese in Beijing, China) population, as reported in dbSNP database.

In the multivariate logistic regression models (Table 3), each of the variant genotypes of *LIG4* rs1805388 was associated with a significantly increased risk of gliomas compared to the wild-type CC genotype (adjusted OR, 1.62; 95% CI, 1.20-2.18 for CT and adjusted OR, 3.27; 95% CI, 1.87-5.71 for TT, respectively). Similarly, compared with the common homozygous genotype, carriers with *XRCC4* rs1805377 homozygous variant genotype showed a significantly increased risk of gliomas (adjusted OR, 1.77; 95% CI, 1.12-2.80 for GG). Furthermore, the association for allele variants were dose dependent for each locus (Trend test: *P* < 0.001 and *P* = 0.030 for rs1805388 and rs1805377, respectively).

**Table 3:** Associations between candidate genes and glioma risk

**Genotype**	**Cases, n(%)**	**Controls, n(%)**	**OR (95% CI)**^**a**^	***P***_**trend**_^**b**^	***P*value for HWE test**
*XRCC3*: rs861539 (T241M)					
CC	336 (87.5)	340 (88.5)	1.00	0.835	0.165
CT	47 (12.2)	41 (10.7)	1.15 (0.73-1.79)		
TT	1 (0.3)	3 (0.8)	0.33 (0.03-3.22)		
*XRCC3*: rs1799794 (−4541G>A)					
GG	100 (26.0)	108 (28.1)	1.00	0.838	0.271
GA	201 (52.4)	181 (47.1)	1.09 (0.78-1.53)		
AA	83 (21.6)	95 (24.8)	0.86 (0.57-1.28)		
*XRCC3:* rs1799796 (A17893G)					
AA	178 (46.4)	171 (44.5)	1.00	0.231	0.406
AT	173 (45.0)	165 (43.0)	0.97 (0.72-1.31)		
TT	33 (8.6)	48 (12.5)	0.64 (0.39-1.04)		
*BRCA2*: rs1799943 (−26G>A)					
GG	158 (41.2)	180 (46.9)	1.00	0.149	0.722
GA	186 (48.4)	168 (43.7)	1.22 (0.90-1.64)		
AA	40 (10.4)	36 (9.4)	1.22 (0.74-2.01)		
*BRCA2*: rs15869 (3' UTR)					
AA	213 (55.5)	220 (57.3)	1.00	0.646	0.375
AC	143 (37.2)	137 (35.7)	1.05 (0.78-1.42)		
CC	28 (7.3)	27 (7.0)	1.04 (0.59-1.83)		
*RAG1*: rs2227973 (R820K)					
GG	129 (33.6)	134 (34.9)	1.00	1.000	0.586
GA	200 (52.1)	190 (49.5)	0.96 (0.70-1.30)		
AA	55 (14.3)	60 (15.6)	0.83 (0.54-1.29)		
*XRCC5*: rs1051685 (3' UTR)					
AA	313 (81.5)	326 (84.9)	1.00	0.232	0.808
AG	69 (18.0)	56 (14.6)	1.24 (0.84-1.82)		
GG	2 (0.5)	2 (0.5)	1.00 (0.37-2.68)		
*LIG4: rs1805388 (T9I)*					
CC	163 (42.4)	222 (57.8)	1.00	**< 0.001**	0.659
CT	172 (44.8)	142 (37.0)	**1.62 (1.20-2.18)**		
TT	49 (12.8)	20 (5.2)	**3.27 (1.87-5.71)**		
*XRCC4: rs1805377 (Splice Site)*					
AA	179 (46.6)	195 (50.8)	1.00	**0.030**	0.454
AG	143 (37.2)	153 (39.8)	0.96 (0.71-1.30)		
GG	62 (16.2)	36 (9.4)	**1.77 (1.12-2.80)**		
*ATM*: rs189037 (−111G/A)					
GG	140 (36.5)	125 (32.5)	1.00	0.487	0.070
GA	186 (48.4)	203 (52.9)	0.78 (0.57-1.07)		
AA	58 (15.1)	56 (14.6)	0.88 (0.57-1.37)		

^a^Adjusted for age, gender, smoking and drinking status.

^b^False Discovery Rate (FDR) corrected P-value.

*P* < 0.05 for bold significances.

### Interaction between smoking and genetic factors

We further evaluated the interaction between the *LIG4* rs1805388 and *XRCC4* rs1805377 polymorphisms and tobacco smoking with respect to the risk of gliomas. We detected a significant additive (*P*_interaction_ = 0.013) and multiplicative interaction (*P*_interaction_ = 0.046) effect between *LIG4* rs1805388 and tobacco smoking for the development of gliomas. Compared with never-smokers carrying the wild-type genotype of LIG4 rs1805388, those ever-smokers with variant-containing genotype of LIG4 rs1805388 polymorphism had a significantly increased risk to develop gliomas (adjusted OR, 1.67; 95% CI, 1.11-2.50) (Table 4).

**Table 4:** Risk of glioma associated with genotypes by smoking status

**Genotype**	**Smoking**	**Cases, n(%)**	**Controls, n(%)**	**OR (95% CI)**^**a**^	***P***_**interaction**_^**b**^
*LIG4*: rs1805388					
CC	No	107 (27.9)	122 (31.8)	1.00	**0.013/0.046**
CT/TT	No	121 (31.5)	96 (25.0)	1.39 (0.96-2.01)	
CC	Yes	56 (14.6)	100 (26.0)	0.62 (0.41-0.94)	
CT/TT	Yes	100 (26.0)	66 (17.2)	**1.67 (1.11-2.50)**	
*XRCC4*: rs1805377					
AA	No	120 (31.2)	117 (30.5)	1.00	0.536/0.886
AG/GG	No	108 (28.1)	101 (26.3)	0.99 (0.68-1.44)	
AA	Yes	59 (15.4)	78 (20.3)	0.70 (0.46-1.07)	
AG/GG	Yes	97 (25.3)	88 (22.9)	1.02 (0.69-1.50)	

^a^Adjusted for age, gender and drinking status.

^b^*P* for additive interaction/*P* for multiplicative interaction.

### Combined analysis of multiple SNPs

The LIG4–XRCC4 complex plays a fundamental role in DNA non-homologous end-joining and is present in all eukaryotes. It has been demonstrated that XRCC4 can stimulate LIG4 activity and is required to stabilize LIG4. Thus, we estimated the combined effect of *LIG4* and *XRCC4* genes on glioma risk. As shown in Table 5, 35.4% of the cases and 19.3% of the controls had variant genotypes at both loci (*LIG4* rs1805388 CT+TT and *XRCC4* rs1805377 AG+GG). In comparison with the reference combination of *LIG4* rs1805388 CC and *XRCC4* rs1805377 AA, the combination of the *LIG4* rs1805388 CT+TT genotype together with *XRCC4* rs1805377 AG+GG genotype was found to be significantly associated with glioma (adjusted OR, 2.22; 95% CI, 1.49-3.30). Furthermore, significant more-than-multiplicative (0.005) and more-than additive (0.009) gene-gene interactions of these two loci (*LIG4* rs1805388 CT+TT and *XRCC4* rs1805377 AG+GG) were found in relation to the risk of gliomas.

**Table 5:** **Interaction of*LIG4*rs1805388 and*XRCC4*rs1805377 on risk of glioma**

***LIG4*rs1805388**	***XRCC4*rs1805377**	**Cases, n(%)**	**Controls, n(%)**	**OR (95% CI)**^**a**^	***P***_**interaction**_^**b**^
CC	AA	94 (24.5)	107 (27.9)	1.00	**0.005/0.009**
CC	AG/GG	69 (18.0)	115 (29.9)	0.73 (0.48-1.09)	
CT/TT	AA	85 (22.1)	88 (22.9)	1.17 (0.78-1.75)	
CT/TT	AG/GG	136 (35.4)	74 (19.3)	**2.22 (1.49-3.30)**	

^a^Adjusted for age, gender, smoking and drinking status.

^b^*P* for additive interaction/*P* for multiplicative interaction.

## Discussion

Accumulating evidence demonstrates that the DSB repair pathway plays a critical role in repairing double-strand breaks caused by a variety of exposures. Although genetic variants in DSB repair pathway genes are considered as potential risk factors for various cancers, less evidence exists as to the potential role of the DSB repair pathway genes polymorphisms on glioma susceptibility. To our knowledge, this study is the first to provide a comprehensive evaluation of the relationship between polymorphisms in both NHEJ and HR pathway genes and susceptibility to gliomas. On the basis of our analysis of 384 controls and 384 glioma patients, we observed that one splice-site SNP in *XRCC4* (rs1805377, IVS7-1A>G, splice-site) and one non-synonymous SNP in *LIG4* (rs1805388, Ex2 +54C>T, Thr9Ile) are associated with the increased susceptibility to gliomas in a Chinese population.

Previous researches on the function of the *XRCC4* rs1805377 and *LIG4* rs1805388 polymorphisms have been informative in understanding the potential roles of these two polymorphisms in the development of gliomas. The *LIG4* rs1805388 polymorphism results in a nonsynonymous amino acid change from threonine to isoleucine at the N-terminal of the LIG4 protein that is essential for its activity [15]. Two linked polymorphisms rs1806389 (T9I) and rs1805388 (A3V) in the N-terminal of LIG4 mildly but reproducibly reduce adenylation and ligation activities (2-3fold) [16] and increase the hydrophobic nature of this region of the protein [17]. The *XRCC4* rs1805377 polymorphism in intron 7 may have functional significance since the nucleotide change potentially abolishes an acceptor splice site at exon 8 [18,19].

Gene-gene interaction was also studied since XRCC4 and LIG4 proteins form a tight and specific complex that catalyzes ligation of processed DNA ends. Although LIG4 interacts with the coiled-coil region of human XRCC4 via the region that lies between the two C-terminal BRCT domains [15,20,21], the combined analysis of multiple SNPs revealed that *LIG4* rs1805388 which causes a nonsynonymous amino acid change at the N-terminal of the LIG4 protein and *XRCC4* rs1805377 interacted to modulate the risk of gliomas as a joint effect. Tseng et al. [22] performed a gene-gene interaction analysis which revealed that polymorphisms in the *XRCC4* (rs1805377) and *LIG4* (rs1805388) genes interacted to modulate the risk of lung cancer (adjusted OR, 8.75) and demonstrated that *LIG4* rs1805388 and *XRCC4* rs1805377 polymorphisms are linked significantly with high fractional allelic loss (FAL), an indicator of genomic instability. Taken together, considering the functional relevance of these two proteins, an individual SNP or combinations of these two SNPs may change the activity of the LIG4-XRCC4 complex and pose a substantial influence on the development of gliomas.

Overwhelming evidence indicates that our findings are biologically plausible. NHEJ is a multistep process initiated by the XRCC5/XRCC6 dimer (also known as Ku80/Ku70) which immediately binds to both broken ends of DNA and recruits the DNA-dependent protein kinase catalytic subunit (DNA-PKcs) forming the trimeric DNA-PK holoenzyme [23,24]. Finally, the LIG4–XRCC4 complex in vivo carries out the ligation step to complete repair [10]. XRCC4 serves as a multipurpose partner for the LIG4 protein, facilitating LIG4 stability and stimulating LIG4 adenylation [21]. Consistent with the need for effective repair of DSBs by NHEJ, *XRCC4*- or *LIG4*-deficient mouse fibroblasts exhibit marked sensitivity to ionizing radiation, growth defects and premature senescence [25,26]. The deficiency of DSB repair has led to significant improvements in radiation sensitization of gliomas [27]. Furthermore, *XRCC4* or *LIG4* null mice die in late embryogenesis accompanied by defective lymphogenesis and massive apoptotic cell death of newly generated postmitotic neurons [28,29]. Many studies in the past have shown that the deficiency of *LIG4* or *XRCC4* in animals can lead to increased rates of neoplastic transformation. Although loss of p53 expression rescues neuronal death and embryonic lethality, *XRCC4* or *LIG4/p53* double-null mice routinely succumbed to RAG-dependent pro-B lymphomas with translocations/amplifications of c-myc and IgH loci [28,29]. Nijnik et al. found that LIG4^Y288C^ mice (a mouse model for human LIG4 syndrome) exhibit multiple defects in lymphocyte development and a hypomorphic *LIG4* mutation can confer strong predisposition to lymphoid malignancies [30]. In addition to tumors of the immune system, Sharpless et al. demonstrated that *LIG4* haploinsufficiency with decreased NHEJ activity contributes to development of soft tissue sarcomas that possess clonal amplifications, deletions and translocations [31]. A defective DNA double-strand break repair pathway in the nervous system can also lead to brain tumors. Lee et al. demonstrated that *LIG4/p53* double-null mice can develop medulloblastoma [32]. Consistent with this notion, *XRCC4/p53* doubly deficient in nestin-expressing neuronal progenitor cells can lead to early onset of neuronally differentiated medulloblastomas [33]. Significant down-regulation of *XRCC4* was found in grade II, III, IV of astrocytoma compared to normal brain tissues and decreased expression of *XRCC4* was significantly associated with a poor prognosis (*P* < 0.05) [34]. These studies raise the possibility that decreased *LIG4* or *XRCC4* activity plays a role in human carcinogenesis.

Since tobacco is a well-confirmed inducer of DNA damage, in particular DSBs [35], we performed stratified analysis to estimate the interaction between the genotypes and smoking status. As shown in Table 4, *LIG4* rs1805388 were associated with an increased risk of gliomas among smokers under a dominant model. Our data indicated the presence of an interaction between the NHEJ pathway genes and smoking status. In addition, smokers with less efficient DSB repair capacity may be more likely to develop gliomas.

Currently, the number of genome-wide association studies (GWAS) has been growing rapidly, leading to the discovery of many new variants associated with complex diseases. Two recent genome-wide association studies (GWAS) of risk of glioma in European populations did not identify an association between the *XRCC4* rs1805377 and *LIG4* rs1805388 polymorphisms and glioma risk [36,37]. There are several possible reasons for the contradictory findings between GWA studies and our present study. First, it might be due to genetic heterogeneity (both allelic and locus heterogeneity) in different ethnic populations or the different reporting criteria for a *P* value. Second, the frequencies of XRCC4 rs1805377 and LIG4 rs1805388 polymorphisms and patterns of linkage disequilibrium (LD) are very different in two HapMap populations (CEU and CHB). Thirdly, it could be that the association of this variant may be population-specific and the interaction between genes and environmental factors vary in different human populations. Our results require confirmation in further GWA studies of gliomas in Chinese population.

Our study has several strengths. First, all tested SNPs were in Hardy-Weinberg equilibrium in controls. Second, in this study, a standardized genotyping approach was performed and quality control samples indicated a high degree of reproducibility of the genotyping results. Third, we were able to examine the association between the 2 SNPs and the risk of gliomas in a well-described and racially homogeneous population of the same ethnicity. Moreover, we use a pathway-based approach to estimate the combined effect of *LIG4* and *XRCC4* genes, which may provide enhanced risk assessment. Finally, we used a relatively comprehensive analysis of 10 polymorphisms in 7 candidate genes involved in DNA double-strand break repair pathways. However, our current study has some limitations. First, we were not able to explore the exact biological mechanism of how *XRCC4* rs1805377 and *LIG4* rs1805388 polymorphisms affect the development of gliomas. Second, this study was a hospital-based case–control study; thus, selection bias in the present study may have led to spurious findings. However, the controls and cases were matched on age and sex, which may have minimized selection bias. Third, our findings need to be replicated in other independent studies. Fourth, we did not perform the stratified analysis by glioma grade due to the limited sample size. Therefore, large-scale studies and functional evaluation are warranted to replicate these findings in an independent population that is well-powered to performed stratified analysis by glioma grade.

## Conclusions

In conclusion, of the 10 potential functional polymorphisms investigated here, we provide the first evidence that the *XRCC4* rs1805377 (IVS7-1A>G, splice-site) and *LIG4* rs1805388 (Ex2 +54C>T, Thr9Ile) polymorphisms contribute to the risk of developing gliomas, alone or in combination. This is also the first report to investigate the role of gene-gene and gene-environment interactions of these 10 potential functional SNPs of 7 major NHEJ and HR pathway genes in the development of gliomas. These findings may be helpful in improving our understanding of the etiology of gliomas. *XRCC4* rs1805377 and *LIG4* rs1805388 polymorphisms may be useful susceptibility biomarkers for gliomas and aid in the development of diagnostic strategy to reduce the burden of gliomas. Future larger scale studies with ethnically diverse populations and functional evaluation are needed to confirm our findings.

## Competing interests

The authors declare that they have no competing interests.

## Authors’ contributions

PZhao and PZou participated in collection of data and manuscript preparation. PZou and LZ performed the statistical analysis. WY, CK, YY and TJ provided the samples. PZhao participated in study design and critically revised the manuscript. PZhao and TJ participated in study design and manuscript preparation. All authors read and approved the final manuscript.

## Pre-publication history

The pre-publication history for this paper can be accessed here:

http://www.biomedcentral.com/1471-2407/13/234/prepub
